# Energy Homeostasis in Monotremes

**DOI:** 10.3389/fnins.2017.00195

**Published:** 2017-04-21

**Authors:** Stewart C. Nicol

**Affiliations:** Biological Sciences, University of TasmaniaHobart, TAS, Australia

**Keywords:** echidna, platypus, hibernation, leptin, thyroid, brown adipose tissue, basoendothermy, evolution of endothermy

## Abstract

In 1803, the French anatomist Étienne Geoffroy Saint-Hilaire decided that the newly described echidna and platypus should be placed in a separate order, the monotremes, intermediate between reptiles and mammals. The first physiological observations showed monotremes had low body temperatures and metabolic rates, and the consensus was that they were at a stage of physiological development intermediate between “higher mammals” and “lower vertebrates.” Subsequent studies demonstrated that platypuses and echidnas are capable of close thermoregulation in the cold although less so under hot conditions. Because the short-beaked echidna *Tachyglossus aculeatus*, may show very large daily variations in body temperature, as well as seasonal hibernation, it has been suggested that it may provide a useful model of protoendotherm physiology. Such analysis is complicated by the very significant differences in thermal relations between echidnas from different climates. In all areas female echidnas regulate T_b_ within 1°C during egg incubation. The lactation period is considered to be the most energetically expensive time for most female mammals but lactating echidnas showed no measurable difference in field metabolic rate from non-lactating females, while the lactation period is more than 200 days for Kangaroo Island echidnas but only 150 days in Tasmania. In areas with mild winters echidnas show reduced activity and shallow torpor in autumn and early winter, but in areas with cold winters echidnas enter true hibernation with T_b_ falling as low as 4.5°C. Monotremes do not possess brown adipose tissue and maximum rates of rewarming from hibernation in echidnas were only half those of marmots of the same mass. Although echidnas show very large seasonal variations in fat stores associated with hibernation there is no relationship between plasma leptin and adiposity. Leptin levels are lowest during post-reproductive fattening, supporting suggestions that in evolutionary terms the anorectic effects of leptin preceded the adiposity signal. BMR of platypuses is twice that of echidnas although maximum metabolism is similar. High levels of thyroid hormones in platypuses may be driving metabolism limited by low body temperature. Monotremes show a mosaic of plesiomorphic and derived features but can still inform our understanding of the evolution of endothermy.

## Introduction

The monotremes are the least speciose of the major extant mammal groups: there are roughly 5,500 species of eutherian mammal and 350 marsupial species but only five extant monotreme species and these are restricted to Australia and New Guinea: the platypus (*Ornithorhynchus anatinus*; Grant, 2015), the short-beaked echidna (*Tachyglosus aculeatus*), and three species of long-beaked echidna (*Zaglossus* spp.; Griffiths, 1978; Flannery and Groves, 1998; Nicol, 2015). Unlike all other mammals, which give birth to live young, monotremes lay eggs. Their unusual reproductive biology and various aspects of their anatomy has led to their frequent depiction as primitive mammals, only slightly removed from the “lower vertebrates.” The term “lower vertebrates” with all its overtones of the *scalae naturae* or “Great Chain of Being” dating back to the ideas of Aristotle (Mayr, 1982), is normally applied to the fish, amphibians, and reptiles (Bennett, 1978). The major problem of using the terms “higher” and “lower” in describing taxa is that they are closely linked to the idea that humans and their closest relatives are the goal of a progression toward a higher level of complexity (Diogo et al., 2015). This thinking persists when biologists read phylogenetic trees as ladders of progress or assume that species-poor lineages that appear “early branching” are basal (Omland et al., 2008).

Despite previously expressing reservations about the application of the concepts of “highness” and “lowness” to animals (Darwin, 1854), in the Descent of Man Darwin wrote “The Monotremata are plainly allied to the Marsupials; forming a third and still lower division in the great mammalian series” (Darwin, 1871). The term “lower mammals” continued to be used until relatively recently in the comparative physiology literature to refer to monotremes, marsupials, and some placentals, particularly when discussing thermoregulation (Johansen, 1962). The similarly problematic term “primitive” is often still used to describe the extant monotremes (Omland et al., 2008), but while many aspects of their anatomy and physiology are plesiomorphic it does not follow that this is the case in all aspects of monotreme biology. Egg-laying is clearly plesiomorphic, but the brain of monotremes, particularly the tachyglossids, is comparable in size and complexity to that of eutherian carnivores. Even in the post-cranial skeleton, which is often described as primitive, the monotremes demonstrate mosaic evolution, combining primitive with very specialized features, e.g., retaining a shoulder girdle of a therapsid pattern but possessing a pelvis of therian pattern (Crompton and Jenkins, 1973). From the first physiological investigations, discussion of the physiology of the monotremes has been influenced by the presumption of primitivity in all aspects of their biology.

A distinguishing feature of the “higher vertebrates”—mammals and birds—is endothermy, the maintenance of a high and (relatively) constant body temperature by metabolic means (Bennett and Ruben, 1979). This distinction was integral to the classification of animals proposed by Linnæus, who divided animals into six classes: Mammalia, birds, amphibia, fishes, insects, and worms. The mammals and birds he grouped together as having a heart with two auricles and two ventricles, and warm red blood; the amphibia (which included reptiles) and fishes were grouped together as having one auricle and one ventricle, and cold red blood (Kerr, 1792). In 1803, the French anatomist Étienne Geoffroy Saint-Hilaire decided that the newly described echidna and platypus did not fit in the Linnæan groupings and should be placed in a separate order, the monotremes, intermediate between reptiles and mammals (Geoffroy Sàint-Hilaire, 1803). Much of the debate about the status of the monotremes revolved around their mode of reproduction but the consensus was that they were primitive and imperfect mammals and close to reptiles.

**Figure 1:**
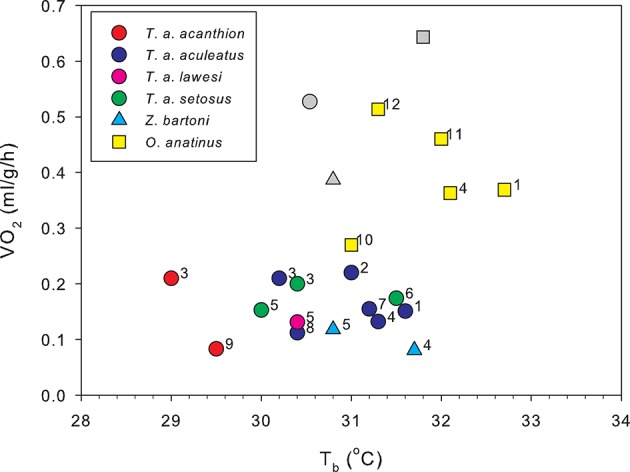
**Metabolic rates plotted against body temperature for resting, unrestrained monotremes at thermoneutrality**. Colored circles show data from four of the echidna subspecies. Gray symbols are metabolic rates calculated for eutherian mammals of the same mass as the respective monotreme means, using a phylogenetic least-squares model based on data from 543 eutherian mammals (Capellini et al., 2010) plotted against the mean T_b_ from each monotreme species. The measurements by Martin (1903) (1), are remarkably close to more recent results although his single platypus was only half grown. Of the other platypus data, point 10 with a lower T_b_ and ${\dot{\text{V}}\text{O}}_{2}$ than the other data, is from a single animal of unspecified mass, whose other physiological responses seem abnormal (Grant and Dawson, 1978), data in point 11 were collected from resting, but not post-absorptive platypuses, and data for point 12 were obtained in the field. The ${\dot{\text{V}}\text{O}}_{2}$ measured for the Western Australian *T. a. acanthion* (9) is much lower than any other for this species. 1, Martin (1903); 2, Schmidt-Nielsen et al. (1966); 3, Augee (1978); 4, Dawson et al. (1979); 5, McNab (1984); 6, Bech et al. (1992); 7, Frappell et al. (1994); 8, Kuchel (2003); 9, Barker et al. (2016); 10, Smyth (1973); 11, Bethge et al. (2001); 12, Frappell (2003).

The first physiological measurements of monotremes reinforced this view. Body temperatures of both Australian monotremes were measured by the Russian explorer turned Australian biologist, Nicholas Miklouho-Maclay: he found the echidna to have a temperature of 28°C and the platypus 24.8°C (Miklouho-Maclay, 1883, 1884). Sutherland (1896) found an average T_b_ for echidnas of 29.4°C, and commenting on his own, and Miklouho-Maclay's results, wrote “…the platypus, therefore, at only 24.8° is almost a cold blooded animal. The only other genus of monotremes, the echidna, carries us a step upwards”. However, he found “an echidna on a cold morning was a low as low as 22°”, while one “in a sack, exposed to fierce midday heat registered 36.6°.” He commented “This is an immense range for a mammal, and suggests a reptilian want of capacity for temperature regulation.” The first measurements of metabolic rate were made by C. J. Martin. Citing Sutherland's work, Martin wrote “Without doubt …monotremes and marsupials present a stage of physiological development intermediate between the fairly accurate homoeothermism of the higher mammals, and the rudimentary indications in this direction …which occur in lower vertebrates.” Martin's paper (Martin, 1903) was “an attempt to locate more precisely the position of the monotremes and marsupials in this ascending scale of physiological superiority to the temperature of the environment.” As well as measuring rectal temperature, Martin measured metabolic rates by gravimetric estimation of CO_2_ production in a range of Australian animals, including a platypus and three echidnas. Martin found a mean rectal temperature for the monotremes of 29.8°C and metabolic rates which are quite close to much more recent measurements (Figure 1).

## Metabolic rate and body temperature

Figure 1 shows quite clearly that the monotremes are all characterized by low T_b_ and metabolic rates, with the platypus having significantly higher basal metabolic rate (BMR) than the echidnas. Depending on the allometric relationship used to calculate the standard eutherian metabolic rates, BMR of the long- and short-beaked echidnas is 25–40% of the corresponding eutherian values, and platypus 70–80% (Dawson et al., 1979; Dawson and Grant, 1980; Capellini et al., 2010; Barker et al., 2016). Can the low metabolic rates of monotremes be attributed to their low T_b_? This would be consistent with the metabolic theory of ecology, which claims that the metabolic rate of an organism is a function of its mass and temperature (Gillooly et al., 2001; Brown et al., 2004; Clarke, 2006). Repeated attempts have been made to explain the BMR differences between birds and mammals, and eutherians and marsupials, in terms of differences in T_b_ (White and Seymour, 2005), and in such comparisons BMR is adjusted to a common T_b_ using appropriate Q_10_ values. Q_10_ provides a useful way to investigate the mechanisms by which metabolism is supressed in individuals, or within a species, in daily torpor and hibernation (Nicol et al., 1992; Geiser, 2004), but using T_b_ adjustment to allow comparisons between taxa seems a fairly meaningless exercise—it is not really clear what the results of such an adjustment tell us. Monotremes are not just “detuned” eutherian mammals: the average eutherian T_b_ is lethal for monotremes (Augee, 1976). In the first attempt to apply this temperature “correction” to monotremes, Dawson and Hulbert (1970) found adjusting the BMR of the echidna to 38°C gave a value close to the allometric prediction for eutherian mammals, and more recently Barker et al. (2016) using a Q_10_ with a constant conductance correction, obtained a similar result. However, as pointed out by Dawson et al. (1979) temperature cannot account for the difference between the monotremes: although the mean T_b_ of resting platypuses is only 1°C higher than the echidnas, BMR is more than twice as high (Figure 1).

The BMR of the platypus and echidnas can be partly explained in terms of the evolutionary trade-off hypothesis: the resting metabolic rate of an organism is the result of a trade-off between resting costs and scope for activity, with the precise level being set by lifestyle (Clarke, 2006). Water has a higher thermal conductivity (2.4 × higher) and specific heat (4,000 × higher) than air, leading to higher rates of heat loss in water, and semiaquatic mammals are also relatively inefficient swimmers (Fish, 2000). Because of these energetic disadvantages semiaquatic eutherian species have a higher BMR than similarly sized terrestrial species (Fish, 2000). The platypus has very dense fur which retains a high insulative value in water, and a number of vascular adaptations which reduce heat loss, but even so at water temperatures below 20°C heat loss of resting platypuses is double that in air at the same temperature (Grant and Dawson, 1978; Bethge et al., 2001). Metabolic rate further increases during foraging activity (Fish, 2000; Bethge et al., 2001), but even when foraging at water temperatures very close to freezing, platypuses maintain their body temperature within the normal range (Grigg et al., 1992), which means that heat loss is being matched by increased heat production. Even in the coldest water platypuses forage on average about 12 h/day (Bethge et al., 2009), and these sustained high levels of energy expenditure have selected for a higher BMR than the terrestrial echidna.

BMR is also influenced by phylogeny (Capellini et al., 2010; Clarke et al., 2010), as is T_b_ (Clarke and Rothery, 2008; Lovegrove, 2012). McNab (1992, 2008) demonstrated that BMR is strongly correlated with diet, and that ant- and termite-eating mammals have a low BMR as well as low T_b_ (McNab, 1984). A more recent analysis has shown that this well accepted relationship between diet and BMR vanishes when T_b_ is included in the model (Clarke et al., 2010), suggesting that the underlying relationship is between diet and T_b_, with BMR responding through its dependence on T_b_. A subsequent analysis of diet and T_b_ patterns in mammals and birds confirmed this strong relationship between T_b_ and diet, with predators of invertebrates having the lowest T_b_ (Clarke and O'Connor, 2014). All the monotremes feed nearly exclusively on invertebrates, although platypuses may occasionally take small fish (Nicol, 2013). Thus, the low T_b_ and BMR of the monotremes can be considered to be the result of their phylogeny and respective ecological niches, although it could be argued that phylogenetic constraints on T_b_ and metabolism may have restricted possible ecological niches. Herbivores consuming grass or leaves have a T_b_ about 2.6°C higher than carnivores taking invertebrate prey (Clarke and Rothery, 2008), and the monotreme line may not have been able to achieve or tolerate higher T_b_ that would have been necessary to occupy other niches. The operative temperature of active short-beaked echidnas and platypuses is about 32°C and the maximum T_b_ recorded in active platypuses is about 34.6°C (Grigg et al., 1992) and in echidnas about 35°C (Brice et al., 2002; Nicol and Andersen, 2002; Nicol et al., 2004) but in both species T_b_ very rarely exceeds 34°C.

Ninety percentage of oxygen consumption at BMR occurs in the mitochondria (Rolfe and Brown, 1997), and while the metabolic differences between reptile and mammal are reflected in differences in total mitochondrial membrane surface area (Else and Hulbert, 1985a), no difference in mitochondrial surface area was seen between the echidna and other mammals (Else and Hulbert, 1985b). Extensive studies on mitochondrial function led Hulbert and Else (2005) to propose the membrane pacemaker theory of metabolism: metabolic rate is determined by the activities of membrane-bound proteins that are either directly or indirectly associated with the energy-consuming processes of cells; the activities of membrane-bound proteins such as transporters, channels, and receptors are influenced by different membrane environments; and the composition of membranes (such as changes in fatty acid or acyl composition) and concomitant changes in membrane properties is the common underlying factor underpinning change in the metabolic rate of animal. However, mitochondrial proton leak is greater in marsupials than in eutherians, although marsupials have lower BMRs, and thus the differences between mammalian taxa do not seem to be explained by mitochondrial proton permeability (Polymeropoulos et al., 2011).

BMR is generally believed to be an indicator of metabolic capacity (White and Seymour, 2005) and although there has been debate about how the metabolic constraints on life history variables relate to BMR (Johnson et al., 2001; Mueller and Diamond, 2001), in placental mammals energy expenditure on reproduction is positively correlated with energy expended on maintenance. Thus, high-maintenance species harvest more energy and expend more on reproduction than low-maintenance species (McNab, 2002) while a low BMR optimizes longevity. The maximum lifespan of the short-beaked echidna is at least 50 years (Hulbert et al., 2008), while female platypuses have a lifespan of up to 21 years (Grant, 2004). An eastern long-beaked echidna at Taronga Zoo was at least 53 when she died. There are no direct measurements of field metabolic rates (FMR) of platypuses, but FMR of short-beaked echidnas measured using the doubly labeled water method was 2.7 times the BMR (Green et al., 1992; Schmid et al., 2003). ${\dot{\text{V}}\text{O}}_{2}$ max for echidnas estimated from treadmill exercise (Edmeades and Baudinette, 1975) and from maximal rewarming rates from hibernation (Nicol and Andersen, 2008), is ~1.44 ml O_2_ g^−1^ h^−1^, 9 times the BMR, but only 28% of the value predicted for wild eutherian mammals of the same mass (Taylor et al., 1981). The highest metabolic rate recorded for platypuses is 1.9 O_2_ g^−1^ h^−1^ when walking on a treadmill (Bethge et al., 2001), 4.2 times BMR, while the highest metabolic rates recorded while foraging in cold water are only 3.2 times BMR (Grant and Dawson, 1978; Bethge et al., 2001). If the reported maximum metabolic rates are corrected to mass independent values using a mass exponent of 0.67 (White and Seymour, 2005) these become 0.36 ml O_2_ kg^−0.67^ min^−1^ for the playpus and 0.35 ml O_2_ kg^−0.67^ min^−1^ for the echidna.

Platypuses occur in permanent freshwater environments in the Australian east, from Cooktown in north Queensland (15°S) to Tasmania (43°S) (Nicol, 2013; Grant, 2015). At Cooktown freshwater river temperatures may reach 31°C (Howley, 2012) and in Tasmania platypuses forage in water at nearly 0°C (Bethge et al., 2003). Mean mass of female platypuses from a north Queensland population was 0.75 ± 0.08 kg and from Tasmania 1.21 ± 0.13 kg (Nicol, 2013). This would normally be considered to be an example of Bergmann's Rule; the platypuses from the colder areas will be bigger to limit heat loss, but it may be better to look at it from the inverse view: platypuses in the warmer areas will be smaller to maximize heat loss. This would be consistent with the heat dissipation limit theory—an upper boundary on total energy expenditure is imposed by the maximal capacity to dissipate body heat and therefore avoid the detrimental consequences of hyperthermia (Speakman and Król, 2010), which will occur at lower ambient temperatures in an endotherm with a low T_b_. Platypuses have a modest ability to sweat (Augee, 1976; Grant and Dawson, 1978) and thus can lose heat when air temperature exceeds T_b_, but during active swimming, when heat production increases by four times over basal (Bethge et al., 2001), the only means of dissipating metabolic heat is by conduction to water. Much smaller body size at the northernmost part of its range is consistent with high water temperatures being an important factor in limiting platypus distribution. Cold water does not appear to be so limiting for platypuses. They have a number of adaptations that minimize heat loss when foraging in cold water: the fur has a high insulation value, higher than that of the polar bear and beaver, and vascular structures in the skin and hind limbs which greatly decrease heat loss (Grant and Dawson, 1978). In a Tasmanian highland lake, platypuses foraged on average 11.9 h/day in summer, and 13.2 h/day in winter when water temperatures frequently approached 0°C (Bethge et al., 2003, 2009). There have been no equivalent studies on platypus in the northern part of their range.

Despite the dramatic differences in adult size between north and south, platypuses from all parts of their range are considered to belong to the same species, although mitochondrial DNA shows two major clades: one from mainland Australia and the other from Tasmania/King Island (Gongora et al., 2012). Echidnas occur from sea level to 1,800 m altitude, and in all parts of Australia, as well as eastern New Guinea, but significant differences in appearance between geographic populations, particularly in the hairiness of the pelage, have resulted in their division into five subspecies (Griffiths, 1978; Augee et al., 2006; Nicol, 2015), although these have not been validated genetically. The most widespread sub-species *T. a. acanthion* which occurs throughout the arid zone in all mainland states and the Northern Territory has long spines and very sparse bristly fur. *T. a. aculeatus*, the sub-species from which the echidnas were first described (Shaw, 1792) occupies the coastal temperate zones in south-east Queensland, New South Wales, Victoria and South Australia. The Tasmanian and Flinders Island subspecies (*T. a. setosus*) has soft thick fur which may completely hide the spines, and was initially believed to be a separate species from mainland echidnas (Nicol, 2015). On temperate Kangaroo Island, the sub-species (*T. a. multiaculeatus*) has very long fine pelage obscured by long, thin spines. The northern sub-species, (*T. a. lawesii*) has long stout spines and thick fur and was first described from New Guinea, but Griffiths (1978) suggests that echidnas from tropical northern Australia also belong to this subspecies.

**Figure 2:**
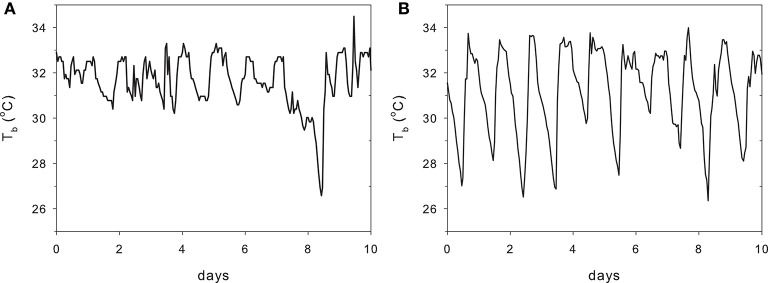
**Body temperature of two adult male echidnas over 10 days in November at (A)** Lovely Banks in Tasmania and **(B)** Stanthorpe in south-eastern Queensland (redrawn from Grigg et al., 2004). Long term average November air temperatures at Lovely Banks: min 7.4°C, max 19.7°C; Stanthorpe min 12°C, max 25.3°C. T_b_ of the Tasmanian echidna drops below 30°C once in these 10 days, whereas in the Queensland echidna this occurs nearly every day. Normal daily variation in the Tasmanian echidna: 3°C, Queensland 6°C.

Augee (1978) found that the conductance of the *T. a. acanthion* was 1.7 times that for *T. a. setosus*. Figure 1 shows metabolic rates and T_b_ measurements for all named subspecies. The two points for *T. a. acanthion* were derived from two geographically distant populations, central Queensland (3) and south-west Western Australia (9), but they both show a very low T_b_ at thermoneutrality, although the metabolic rates are very different. When ambient temperature was reduced from 20°C to 5°C over 58 days, T_b_ of *T. a. acanthion* dropped to 23°C (Augee, 1978). Generally, echidnas from the warmer parts of Australia seem to have more variable T_b_ when active than echidnas from cooler climates. Figure 2 shows T_b_ records over 10 days in November from echidnas in Tasmania (a) and south-east Queensland (b). November is the time of maximum foraging and weight gain for males following the mating period (Kuchel, 2003; Nicol and Morrow, 2012). Echidnas in both locations show daily variations in T_b_ related to activity, rather than T_a_ (Grigg et al., 2004; Nicol et al., 2004), but in Tasmania T_b_ only drops below 30°C following several days of inactivity, whereas at the warmer site the pattern resembles daily torpor. For Tasmanian females the daily T_b_ range of non-lactating individuals in November was 3.1 ± 0.7°C, similar to the males, but lactating females had a significantly greater daily variability (4.8 ± 1.0°C) (Schmid et al., 2003).

Heterothermia appears to be one means whereby echidnas can survive in hot environments: an animal with low initial T_b_ takes longer to reach dangerous levels. Despite this it has been difficult to reconcile the ability of echidnas to survive for many hours at ambient temperatures exceeding T_b_ (Brice et al., 2002) with their apparent inability to use evaporative cooling (Augee, 1976), but a recent study shows echidnas have some capacity to increase evaporative water loss (Barker et al., 2016).

## Hibernation

Many birds and mammals temporarily abandon homeothermic endothermy during times of cold exposure, food shortage or drought, and use the energy minimizing strategies of daily torpor and hibernation (Ruf and Geiser, 2015). The short-beaked echidna is the only one of the monotremes to use these strategies, but the different geographic sub-species vary in their use of torpor and hibernation. Echidnas in all parts of their range show a reduction in activity at about the same time of the year (Nicol and Andersen, 1996; Morrow et al., 2009), but whether they are able to show extended periods of hibernation appears at least partly to depend on the environmental temperature.

**Figure 3:**
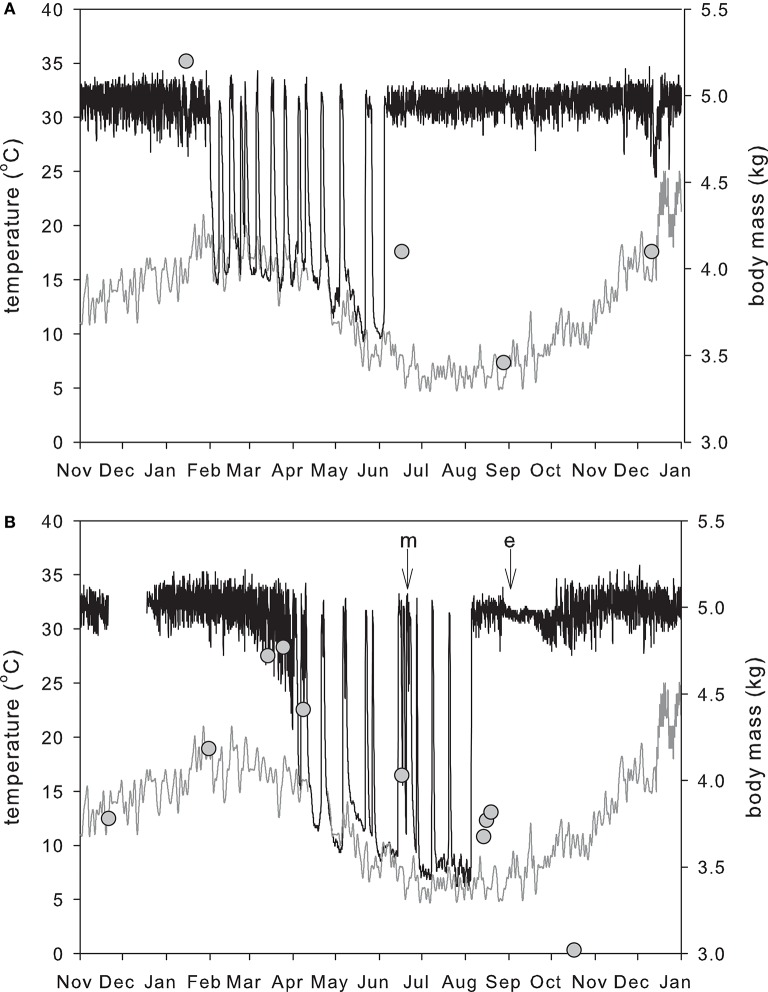
**Annual pattern of body temperature and mass in a reproductively active male (A)** and female **(B)** in Tasmania in the same year. Black line: body temperature; gray line: soil temperature at 20 cm measured at a Bureau of Meteorology station 2 km from the field site; circles: body mass. The male entered hibernation in summer (Feb 1) after building up fat reserves in spring and early summer. The female reached maximum mass and entered hibernation much later (April 4). As in other deep hibernators, hibernation is broken by periodic arousals, although, unlike most other hibernators, echidnas may move to another location during these euthermic periods (Nicol et al., 2011). The male ended hibernation in early winter (June 4) and was found mating with the female on June 17 (m on panel **B**). The pregnant female then re-entered hibernation, and her final arousal from hibernation was on August 5. Shortly after this she entered a nursery burrow and laid an egg (e). Incubation of the egg takes 10–11 days, during which T_b_ remains very stable (Beard et al., 1992; Nicol and Andersen, 2006) and in Tasmania the female typically stays in the burrow with the young for 23–48 days before leaving it in a plugged burrow while she forages (Morrow and Nicol, 2012). When she first emerges from the nursery burrow her body mass is at its lowest. Males reach their minimum mass at the end of the mating period.

**Figure 4:**
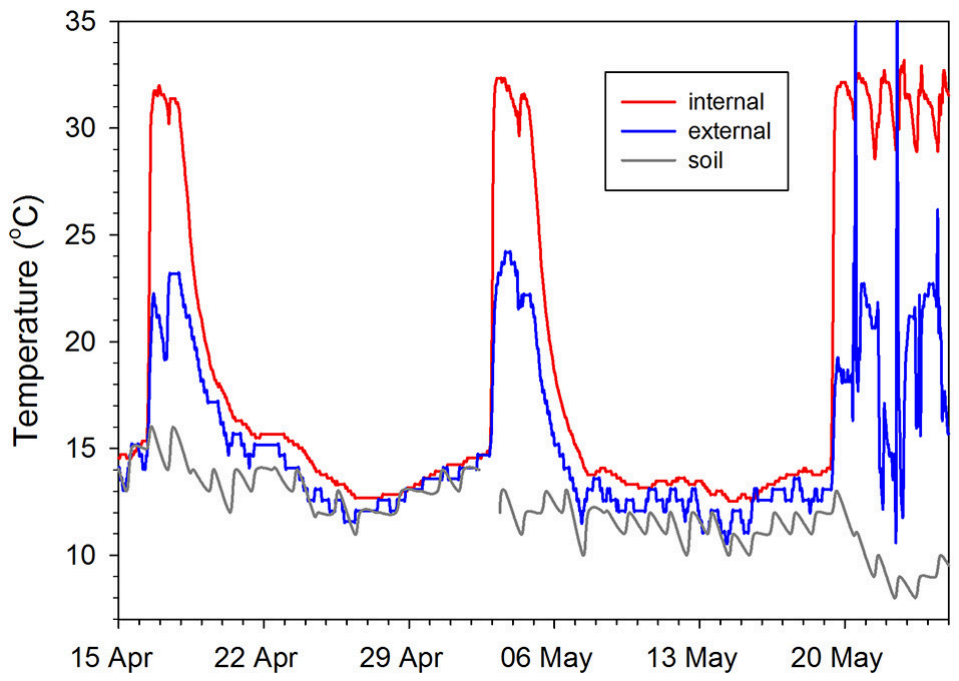
**Relationship between internal, external, and soil temperatures for a male echidna during two periodic arousals and final emergence from hibernation**. Internal temperature (T_b_) was measured by an implanted datalogger (Onset Computer Corporation, Stowaway Tidbit) in the peritoneal cavity, external temperature from a logger (Thermochron iButtons, DS1922L; Maxim/Dallas Semiconductor, TX, USA) attached to a tracking transmitter, which was glued to the spines. Soil temperature was measured at 20 cm depth at a Bureau of Meteorology station 2 km from the field site. Redrawn from Nicol et al. (2008).

At Stanthorpe in Queensland hibernation occurred in 9 out of 15 echidna-years of recording (Kuchel, 2003), whereas at Lovely Banks in Tasmania all echidnas hibernated every year (Nicol and Andersen, 2002). The greater variability in active T_b_ and use of hibernation mean that in echidnas in warm climates it is difficult to distinguish between torpor and non-torpor (Kuchel and Grigg, 2003). Kangaroo Island echidnas showed reduced activity from April to August, but this varied greatly between individuals, and within individuals from year to year (Rismiller and McKelvey, 1996). Some Kangaroo Island echidnas showed several bouts of hibernation, with T_b_ profiles similar to “classical hibernators” and a minimum T_b_ of 11.8°C, while other echidnas in the same area did not hibernate. In the cooler climates of Tasmania and the Australian Alps (Beard et al., 1992) the hibernation period is very distinct. Figure 3 shows a male Tasmanian echidna entering hibernation at the warmest time of the year. When they have built up sufficient fat reserves, echidnas reduce their activity (Sprent et al., 2012), and dig into the soil, and T_b_ falls until it is with 0.5–1.0°C of substrate temperature (Nicol and Andersen, 2002; Figure 4). The factors that determine the equilibrium T_b_ can be seen by rearranging the familiar Scholander-Irving model (Nicol et al., 2008):

(1) $$\begin{array}{c} T_{b}=T_{a}+\frac{\dot{V}O_{2}}{C} \end{array}$$

i.e., T_b_ falls to a temperature dependent on ambient temperature plus an amount determined by the ratio of hibernating metabolic rate to conductance. This relationship only holds for thermoconforming animals above the lower set point (Geiser, 2001; Nicol and Andersen, 2008). If T_b_ drops below the set point, most hibernators increase heat production, which is energetically expensive (Geiser, 2004). Echidnas arouse and move to a warmer area which also represents an energetic penalty and increases the chance of predation. Metabolic rate in hibernating echidnas is about 12% of the normal resting value, and at low T_b_ is relatively independent of T_b_ (Nicol and Andersen, 1993), while the conductance during cooling is the same as in cold exposed non-hibernating echidnas (McNab, 1984; Nicol and Andersen, 2007a). The minimum T_b_ recorded from a hibernating echidna is 4.5°C (Nicol et al., 2008), which seems to be the lower set point. Because cooling takes several days (Figure 4), daily torpor with a stable torpid T_b_ is clearly not an option for echidnas.

As in other hibernators (Geiser et al., 1990), hibernation bout length increases as T_b_ falls (Nicol and Andersen, 2000), and echidnas seem unable to maintain a prolonged hibernation bout when T_b_ is above 17°C. In the record shown in Figure 3, there were several days of very cold nights, cooling the soil, and allowing the male to enter hibernation on February 1. Grigg et al. (2004) have described this behavior as using cold as a resource, i.e., taking advantage of the cold to cool down and enter torpor, thus saving energy. The female, which had not accumulated sufficient fat reserves, did not enter hibernation, but showed only a brief fall in T_b_ to 28°C. Echidnas show behavioral thermoregulation during hibernation; early in the hibernation season echidnas prefer to hibernate in cool areas, while during the coldest months they may move to warmer hibernacula, giving a preferred hibernating T_b_ in the range 7–9°C (Nicol and Andersen, 2007a). This is well above the minimum recorded T_b_ and apparent lower set point of 4.5°C, but may represent a balance between maximizing bout length and thus reducing energetically expensive periodic arousals, and maintaining a safety margin to reduce cold induced arousals. Unlike most hibernators, echidnas do not dig or construct a hibernaculum. Instead they may use existing rabbit or wombat burrows, hollow logs, or dig under tree stumps or into grass tussocks, piles of bark or leaves (Wilkinson et al., 1998) or simply burrow into the substrate, and are often more exposed to ambient conditions.

There is no evidence of hibernation or daily torpor in the long-beaked echidna or the platypus. Captive long-beaked echidnas showed a daily variation of T_b_ of 2–4°C, with a modal T_b_ of 31°C, when short-beaked echidnas in the same pen showed both torpor and hibernation (Grigg et al., 2003). Free-ranging platypuses in the southern Alps monitored maintained a T_b_ close to 32°C throughout the winter (32.1 ± 0.8°C, range 29.2–34.6°C) (Grigg et al., 1992).

Recently Nowack et al. (2016) have demonstrated an increased use of torpor by short-beaked echidnas after fire, and argue that torpor may be an important contributor to survival during natural disasters. Turbill et al. (2011) have shown that hibernation is associated with higher rates of overwinter and annual survival than non-hibernators. This higher survival appears to be due not only to avoidance of sub-optimal environmental conditions (which could include wildfire), but to reduced predation. This further demonstrates that the benefits of torpor extend beyond energy conservation in cold climates (Geiser and Brigham, 2012). The increased survival of hibernating species is linked with the coevolution of traits indicative of slow life histories (Turbill et al., 2011). The limited amount of data from long-beaked echidnas (non-hibernators) suggest very similar life history traits to short-beaked echidnas (hibernators), but as the evolution of slow life histories appears to be related to survival, rather than hibernation *per se* (Turbill et al., 2011) this suggests high rates of survival in long-beaked echidnas. Unfortunately, the echidna fossil record is very sparse and incomplete so that echidna origins are the subject of considerable debate (Phillips et al., 2009; Musser, 2013; Simon, 2013). Short-beaked echidnas appear in the fossil record in the Pleistocene, before which long-beaked echidnas predominated (Musser, 2013), and it seems likely that the slow life history of short-beaked echidnas has not co-evolved with torpor and hibernation, but was a pre-existing tachyglossid trait, which facilitated the expression of torpor and hibernation in this species.

## Reproduction and energetics

The unusual timing of hibernation in echidnas is clearly related to reproduction: in Tasmanian echidnas, in which hibernation appears obligatory, the time from hatching of the young to weaning is about 150 days, at which time the young weighs about 1.5 kg (Morrow and Nicol, 2012). This relatively slow growth rate of the young and an apparent increase in heterothermy by the mother means that daily energy expenditure of females in mid-lactation was not measurably higher than of non-lactating females at the same time (Schmid et al., 2003). As in other seasonal breeders, male echidnas show testicular involution after the breeding season, presumably as an energy saving measure (Griffiths, 1978; Morrow et al., 2016). In order for the young to be weaned before the females enter hibernation, mating must occur in winter but the very large size of the testes (about 1% of body mass at the beginning of breeding) and the low metabolic rate means that, unlike all other hibernators, testicular recrudescence in Tasmanian echidnas occurs before entry into hibernation (Morrow et al., 2016). The very high competition between males for females selects for early arousal by males, which then seek out females, which are still hibernating (Figure 3). Morrow et al. (2015) found that all females that mated prior to July 27 re-entered hibernation, including females that were pregnant. Five of these were monitored; four re-entered hibernation for relatively short periods (3–13 days) but one hibernated for 50 days, showing 4 periodic arousals. Pregnant females that reentered torpor did so no more than 5 days after fertilization, when the embryo would probably be no later than the blastocyst stage (Werneburg and Sánchez-Villagra, 2011; Ashwell, 2013b), and it appears that there is no significant development of the embryo during torpor, as the gestation period is extended by a day for every day in torpor (Nicol and Morrow, 2012). In some respects this is similar to embryonic diapause in marsupials and some eutherian mammals (Lopes et al., 2004), although it is controlled by temperature rather than hormonally. Hibernation during pregnancy is quite unusual; torpor and reproduction have been widely viewed as mutually exclusive but torpor during pregnancy has now been observed in monotreme, marsupial, and eutherian mammals (McAllan and Geiser, 2014). In the majority of species these torpor bouts are daily events lasting a few hours, rather than the extended deep hibernation seen in some female echidnas, however Willis et al. (2006) recorded deep multiday torpor (i.e., hibernation) of up to 5.6 days in pregnant hoary bats (*Lasiurus cinereus*), which enabled parturition to be delayed in unfavourable weather. Similarly, female echidnas that are mated very early, thus benefitting from mating with the fittest males, re-enter hibernation, delaying egg-laying until conditions are more favourable, and ensuring that that maximum growth rate of the young coincides with the period of greatest ecosystem productivity (Nicol and Morrow, 2012; Morrow et al., 2015).

An earlier observation in which a pregnant Kangaroo Island echidna entered torpor only 2 days before egg-laying (Geiser and Seymour, 1989) appears to be different from the hibernation in early pregnancy observed in Tasmanian echidnas. 16 days after capture this female was found to be torpid with a T_b_ of 21°C, but T_b_ and activity had returned to normal 6 h later. An egg shell was found in the cage 2 days later. There is no indication whether the young died accidentally after the egg was laid, or had not survived to this stage. We have 60 records of T_b_ from pregnant Tasmanian echidnas but none of these show any indication of late stage torpor, although several entered torpor after losing the egg or the young. Because the Kangaroo Island echidna young did not survive, it is not clear whether what happened was a stress response of a captive animal, or normal physiological behavior in a sub-species which shows numerous differences from eastern echidnas.

Throughout Australia echidna mating occurs at approximately the same time (June–September), although it appears to be slightly earlier in more southern populations (Morrow et al., 2009). This is in contrast to platypuses, which do not hibernate, and in which breeding begins earlier in more northern populations (Nicol, 2013). Data are only available from a small number of Australian locations, but it appears that in more northern parts of eastern Australia echidna lactation durations are similar to those in Tasmania (Beard et al., 1992; Beard and Grigg, 2000), whereas on Kangaroo Island (South Australia) (Rismiller and McKelvey, 2003) and in Western Australia (Abensperg-Traun, 1989) young are weaned at 204–210 days, although at similar body mass to eastern echidnas. This could be another manifestation of differences in energetics between the geographic sub-species.

A particularly interesting feature of echidna reproduction and thermoregulation is shown in the T_b_ record following egg-laying in Figure 3. While the mother is in the nursery burrow T_b_ is remarkably constant, particularly during the first 10–11 days, which is the egg-incubation period (Nicol and Andersen, 2006; Morrow and Nicol, 2012), where the range is about 1.2°C. This pattern was first observed in echidnas in the Australian Alps (Beard et al., 1992) and subsequently in echidnas in south-east Queensland (Beard and Grigg, 2000).

## Thyroid hormones

As noted above, perhaps the major distinction between birds and mammals and “lower vertebrates” is that all birds and mammals are endothermic, even when inactive. The contribution of the monotremes to our understanding of the evolution of endothermy is discussed later in this review, but whatever the selective process, the acquisition of endothermy appears to be closely linked to thyroid hormones (Little and Seebacher, 2014). The elevated metabolism associated with endothermy in mammals is produced by leaky cell membranes, and thyroid hormones play a key role in regulating metabolic rate by increasing leakiness and thus increasing cellular ATP turnover (Hulbert, 2000). In mammals there is a stoichiometric relationship between oxygen consumption and consumption of thyroid hormones (Tomasi, 1991).

Hulbert (2000) has compiled a comprehensive listing of concentrations of thyroid hormones in vertebrate plasma and I have drawn heavily on his review in this section. Birds and eutherian mammals have much higher circulating levels of thyroid hormones—principally 3′,5′,3,5-l-tetraiodothyronine (thyroxine, T4)—than “lower vertebrates”. In adult reptiles, total plasma thyroxine (TT4) ranges from 1 to 14.5 nmol L^−1^, while in birds (apart from ostriches, which have low values) TT4 is in the range of 15.9–34 nmol L^−1^. In small to medium sized eutherian mammals TT4 is typically is in the range 20–80 nmol L^−1^ (Hulbert, 2000), and TT4 of active echidnas is 15 nmol L^−1^ (Hulbert and Augee, 1982; Nicol et al., 2000), at the low end of the normal range for eutherian mammals and consistent with a low metabolic rate. The only significantly lower TT4 for an adult small mammal comes from a poikilothermic rodent, the naked mole-rat (*Heterocephalus glaber*), with a TT4 of 5 nmoL^−1^, which increases to 7 nmol L^−1^ during cold exposure (Buffenstein et al., 2001).

By contrast with the values for echidnas, TT4 in adult platypuses is high (64 nmol L^−1^) (Hulbert and Grant, 1983), at the upper end of the range for eutherian mammals, and presumably associated with a BMR that is two and a half times that of echidnas. TT4 levels in platypuses did not vary significantly with season, and similarly in active echidnas there was no difference between summer and winter values, but plasma levels of all thyroid hormones in echidnas fell significantly during hibernation (Nicol et al., 2000; Table 1). This is different from what has been observed in other hibernators. Eutherian hibernators show the lowest levels of thyroid hormones pre-hibernation (Hulbert and Hudson, 1976; Young, 1984; Kwiecinski et al., 1991; Damassa et al., 1995; Tomasi and Stribling, 1996), whereas in echidnas thyroid hormone levels trend down during the pre-hibernation period and reach their lowest during the hibernation period.

**Table 1:** **Plasma thyroid hormone levels in active and hibernating (T_b_ 5–12°C) echidnas**.

**Assay**	**Active**	**Hibernating**
TT4 (nmol L^−1^)	15.2 ± 1.1 (23)	7.47 ± 0.95 (8)
FT4 (pmol L^−1^)	20.2 ± 1.5 (23)	10.7 ± 1.9 (8)
TT3 (nmol L^−1^)	1.64 ± 0.05 (22)	1.09 ± 0.06 (8)
FT3 (pmol L^−1^)	4.61 ± 0.23 (23)	2.76 ± 0.14 (8)

*Values are shown as mean ± SEM. Sample sizes are shown in parentheses*.

In eutherian hibernators thyroid hormone levels, although starting low, rise progressively during hibernation. In ground squirrels TT4, FT4, TT3, and FT3 are higher during hibernation than in active animals (Magnus and Henderson, 1988a,b). In woodchucks (Young et al., 1979) found TT4 and FT4 to be highest in early spring and lowest in summer and autumn, while TT3 and FT3 were highest during hibernation. In black bears all four hormone levels decreased prior to hibernation; free hormones remained low during hibernation but the total levels recovered (Tomasi and Stribling, 1996). In male little brown bats (*Myotis lucifugus*), there is a 5-fold increase in TT4 during the course of hibernation (Damassa et al., 1995) and in females the increase is 8-fold (Kwiecinski et al., 1991).

As thyroid hormones are considered to have a major role in regulating metabolic rate, the first findings of increased levels of thyroid hormones in hibernating rodents were unexpected. Blood levels of thyroid hormones reflect the amount bound to proteins and the balance between the rates of secretion and utilization. Elevated total hormone levels during hibernation have been attributed to greatly reduced clearance rates (Demeneix and Henderson, 1978) and increased levels of binding proteins (Magnus and Henderson, 1988a). Most thyroid hormone circulating in the blood is bound to plasma proteins (99.97% of T4 and 99.7% of T3 in humans; 99.86 and 99.72%, respectively, in euthermic echidnas). In large eutherian mammals, some bats, and many marsupial species, three plasma proteins are involved in this transport: albumin, which in humans binds about 15–20% of T4 and T3; transthyretin (TTR) which binds 10–15% of T4 and T3; and thyroxine binding globulin (TBG), which in humans binds about 70% of T4 and T3 (Mendel, 1989). Adult monotremes possess only two thyroid hormone binding plasma proteins: albumin and a post-albumin globulin (E-TBP); TTR has not been detected in plasma from short-beaked echidnas (*Tachyglossus aculeatus*) either when active or hibernating, from long-beaked echidnas, (*Zaglossus bartoni*), or platypuses (Richardson et al., 1994; Richardson, 2009). Using electrophoresis followed by autoradiography Richardson et al. (1994) found the band caused by binding of radioactive thyroxine to protein in the post-albumin region was less intense in plasma from a hibernating echidna than in plasma from a non-hibernating echidna, indicating a reduction in E-TBP levels during hibernation. By contrast in the bat *M. lucifugus*, although TT4 rises during the course of hibernation, TBG remains at basal levels (Damassa et al., 1995). The differences in patterns of seasonal variation in hormone levels between the echidna and other hibernators may well be related to differences in characteristics and levels of these binding proteins.

## Brain and energetics

Thyroid hormones are essential for nervous system and brain growth and development (Hulbert, 2000), but the brain is separated from the rest of the body by the “blood-brain barrier” which restricts the movement of large molecules (Saunders et al., 1999). Transport of thyroid hormones from the blood to the brain is dependent on TTR, the only thyroid hormone transporting protein made in the brain. TTR is synthesized in the choroid plexus and secreted exclusively into the CSF, transporting thyroid hormones from the blood into the brain and throughout the CSF (Richardson, 2009). Transthyretin synthesis in the choroid plexus is believed to have begun at the stage of the stem reptiles, about 320 Ma, which developed the first traces of a cerebral neocortex (Richardson, 2009), and is synthesized by the choroid plexus of the echidna, the only monotreme in which it has been investigated (Richardson et al., 1994).

The platypus and the echidnas both have large, highly encephalized brains with a six-layered isocortex (neocortex) like all therian mammals, but the two monotreme groups have very different cortical morphology (Ashwell, 2013a). In the platypus the isocortex is lissencephalic (smooth) and thick, while in the echidnas it is gyrencephalic (folded) and thin. The olfactory bulb of the echidna is also gyrified (Ashwell, 2013c).

**Figure 5:**
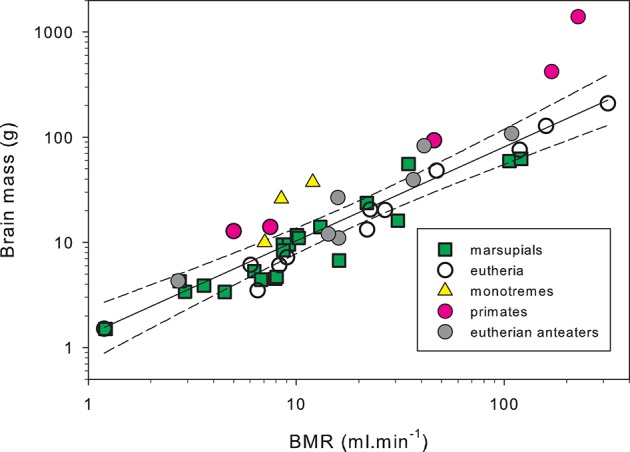
**Brain mass as a function of basal metabolic rate in mammals**. Both BMR and brain mass scale with body mass, and the relationship between brain mass and BMR illustrates the relative investment of energy in the brain. The regression line and 99% confidence limits have been fitted to the data for 12 non-primate non-myrmecophagous terrestrial placental mammals from 11 families and 9 orders (Afrosoricida, Artiodactyla, Carnivora, Chiroptera, Eulipotyphla, Hyracoidea, Lagomorpha, and Scandentia). The two echidnas (*Tachyglossus aculeatus* and *Zaglossus* sp.), and the primates (*Perodictus potto, Nycticebus coucang, Macaca mulatta, Pan troglodytes, Homo sapiens*) lie well above the upper confidence limit. The giant anteater (*Myrmecophaga tridactyla*), the hairy-nosed wombat (*Lasiorhinus latifrons*), and tamandua (*Tamandua tetradactyla*) also lie above the upper confidence limit. Circles: placental mammals, data from McNab and Eisenberg (1989); squares: marsupials, data from Ashwell (2008), Weisbecker and Goswami (2010). Triangles: monotremes, data from McNab and Eisenberg (1989), Nicol and Andersen (2007b). In order of increasing brain mass they are platypus, short-beaked echidna, long-beaked echidna. Solid shading: primates; gray shading: myrmecophages; open symbols: other terrestrial mammals. Body masses have been restricted to a range of 250 to 70,000 g to avoid any effects associated with very low or very high body masses. Metabolic rates were recalculated for the body mass used for brain mass measurement assuming a within-species exponent for metabolic rate of body mass^0.70^ (Kozlowski and Konarzewski, 2005; White and Seymour, 2005; Sieg et al., 2009). To minimize errors associated with this correction, data sets have been restricted to cases where body masses for BMR measurement and body masses used for brain weight were within 30% of each other. Under these circumstances, any errors in BMR correction are trivial. Redrawn from Nicol (2013).

Brain tissue is energetically expensive and during rest it uses nearly an order of magnitude more energy per unit weight than most other somatic tissues (Mink et al., 1981). Analysis of microanatomical features that reflect metabolic activity of the cerebral cortex (capillary volume fraction, and mitochondrial density) suggest that the echidna cerebral cortex has similar levels of metabolic activity to eutherian mammals (Hassiotis et al., 2005). Assuming this, the energy usage of monotreme brains can be estimated from brain mass, using the equations from Hofman (1983), and then adjusting brain metabolic rates from the T_b_ of placental mammals (38°C) to the monotreme value of 32°C. From this the percentage of basal oxygen consumption used by the brain would be about 5.8% for the platypus, 8.5% for the short-beaked echidna, and 9.5% for the long-beaked echidna (Nicol, 2013). Most mammals lie in the range from 2–8% (mean value for 240 mammals is 4.6%) with only primates and cetaceans having values above 8% (Hofman, 1983). However, these estimates depend on several assumptions about the scaling of brain metabolism, and a more direct analysis is provided by simply plotting brain mass as a function of BMR (Figure 5). This graph demonstrates that the echidnas have very large brains relative to their metabolic rate, comparable to the primates.

The expensive tissue hypothesis states that an increase in brain size must be accommodated by an increase in total metabolic rate or by a reduction of the demands of the other expensive organs, such as heart, liver, kidney, and gastrointestinal tract (Aiello and Wheeler, 1995). Thus, it is argued that the relatively large brain sizes of humans and other primates could not have been achieved without a shift to a high-quality diet, allowing a reduction in gut size. It is doubly puzzling then that the short-beaked echidna has a brain of similar size to that of a similar sized eutherian carnivore but a metabolic rate only 30% of the eutherian prediction and has a diet of extremely low energy density and digestibility (Sprent and Nicol, 2016). Echidnas have brain size to BMR relationships similar to those of primates, suggesting that there must be very considerable fitness benefits for the echidnas to maintain such large brains, i.e., the cognitive benefits must outweigh the metabolic costs (Isler and van Schaik, 2006).

The fitness benefits must be considerable for short-beaked echidnas, because the species seems to be specialized to minimize energy expenditure, and many aspects of their ecology and behavior are correlated with small brain size in other mammals. Insectivorous eutherian mammals have smaller brains than carnivores and omnivores (Gittleman, 1986), possibly because a larger brain may be necessary to handle a resource that requires more complex foraging strategies and within primates, larger brain size appears linked to monitoring food sources that vary in space and time (Clutton-Brock and Harvey, 1980). The echidna is the only mammal known to have a gyrified olfactory bulb, probably to expand the number of synaptic glomeruli available for the analysis of the odorant repertoire (Ashwell, 2013c). A total of 186 compounds potentially used in olfactory communication by echidnas have been identified in exudates from the cloaca and base of the spur, including volatile carboxylic acids, aldehydes, ketones, fatty acids, methyl esters, ethyl esters, terpenes, nitrogen- and sulfur-containing compounds, alcohols, and aromatics (Harris et al., 2012). Long chain and very long chain monounsaturated fatty acids, sterols, and sterol esters were identified as the major constituents of solid exudates, some of which have not previously been described from any animal skin gland. There are differences in volatile and non-volatile odorant composition between sexes and individuals but there is no single pheromone—echidnas process a complex suite of chemical signals providing a range of information (Harris et al., 2014, 2016). Echidnas deposit feces in latrines (Sprent et al., 2006), and chemical signals from these are likely to be an important means of communication in echidna populations. Processing this complex olfactory information may have been important in the selection process leading to a high investment in the echidna brain. Platypuses have cervical scent glands on both sides of the neck which produce a musky odor and secretions increase during the breeding season (Grant, 2015), but the olfactory bulb is smaller and unfolded, consistent with olfactory communication being less important in this semiaquatic monotreme.

Large brain size in mammals is also associated with longevity and González-Lagos et al. (2010) suggest that because large brains allow flexible behavioral responses to unusual, novel or complex socioecological challenges they will facilitate a longer reproductive life span. This underlines the need for more behavioral studies of echidnas in their natural habitat across their range (Nicol, 2013). It may be significant that both relative brain size and longevity are greater in the echidna than the platypus. Longevity is also correlated with a low basal metabolic rate (Hofman, 1983; White and Seymour, 2004) and it may be difficult to unravel the causal relationships between metabolic rate, brain size and longevity.

## Leptin and energetics

In eutherian mammals, the peptide hormone leptin has a key role in the regulation of fat reserves. Leptin is synthesized and secreted primarily by adipose tissue, and an increase in adiposity in eutherian mammals is normally associated with a corresponding increase in the synthesis and secretion of leptin by adipocytes, resulting in increased circulating leptin concentrations (Denver et al., 2011). Leptin binds to leptin-specific receptors in the hypothalamus, regulating the production of a range of orexigenic and anorexigenic neuropeptides, and resulting in a decrease in food intake, an increase in metabolic rate, and consequently a loss of adipose tissue (Denver et al., 2011; Florant and Healy, 2012). Although it is frequently claimed that leptin is an adipostat in mammals this been demonstrated only for eutherian mammals; it is not true for the short-beaked echidna (Sprent et al., 2012), and although pharmacological doses of leptin inhibit daily torpor in the marsupial *Sminthopsis macroura* (Geiser et al., 1998) the relationship between adiposity and endogenous plasma leptin has not been investigated in marsupial mammals.

Leptin othologs have now been described for all the major classes of vertebrate (Londraville et al., 2014; Prokop et al., 2014), and the Lep gene has been identified in the genome sequence of the platypus (Denver et al., 2011). The interaction between leptin and the leptin receptor is conserved in terrestrial vertebrates, and in mammals both leptin and its receptor are highly conserved with few variations (Prokop et al., 2012). Sprent et al. (2012) hypothesized that in the echidna as in eutherian hibernators, there would be a strong relationship between adiposity and plasma leptin for most of the year which would change during pre-hibernatory fattening. Instead they found a weak negative relationship between adiposity and plasma leptin. The highest leptin levels were found in both sexes during hibernation and in females during the mating period. As female echidnas return to hibernation after mating, even when pregnant, unless they are further disturbed by males (Harris and Nicol, 2014; Morrow et al., 2015), the high leptin in mating females is most probably also related to hibernation. The lowest leptin levels were recorded from males during the post-reproductive period, when they forage maximally and show their greatest increase in mass (Nicol and Morrow, 2012). Generally high leptin concentrations in echidnas occur during periods when animals show minimal activity, have low body temperatures and do not feed. These results on the echidna are consistent with studies on a variety of non-mammalian vertebrates which have led to the consensus of an ancient role of leptin in regulating food intake and metabolism (Denver et al., 2011; Sprent et al., 2012).

**Figure 6:**
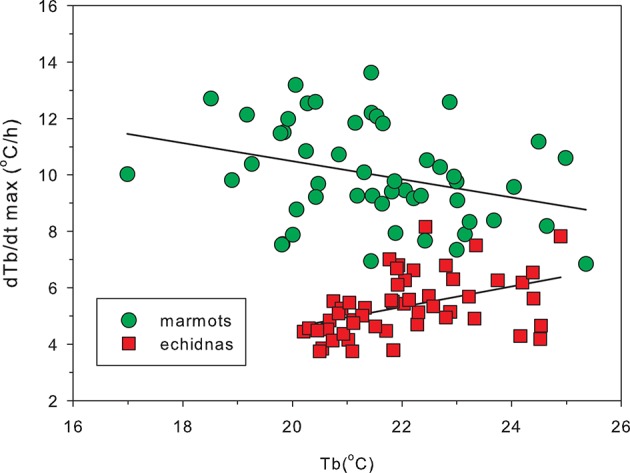
**Peak rates of rewarming from hibernation in marmots (*Marmota marmota*) and echidnas (*Tachyglossus aculeatus*) as a function of body temperature**. Peak rewarming rates were calculated from the first derivative of a sigmoid curve fitted to the rewarming data, and each point represents one rewarming. Because rewarming rate is inversely related to body mass (Geiser and Baudinette, 1990) the figure compares data from hibernators of the same mass (10 marmots, mass 3.39 ± 0.46 kg, mean ± *SD* and 10 echidnas 3.71 ± 0.75 kg). In marmots, which possess BAT, the highest rewarming rates occur at lower T_b_ than in echidnas, although active T_b_ in marmots is 38°C and in echidnas 32°C. The contrasting relationships between peak warming rates and T_b_ reflect a difference between BAT and muscle thermogenesis. Redrawn from Nicol et al. (2009).

Sprent et al. (2012) suggested that the adipostatic role for leptin in eutherian mammals evolved along with BAT-based thermogenesis. In eutherian mammals, leptin increases brown fat (BAT) activation, decreasing metabolic efficiency and increasing heat production, and burning fat stores (Cannon and Nedergaard, 2004). Heat production in BAT results from the activation of mitochondrial uncoupling protein-1 (UCP1), which leads increased proton leak, rather than ATP production. There is no evidence for BAT thermogenesis in marsupials, and no evidence for BAT in monotremes (Oelkrug et al., 2015). Molecular phylogeny of UCP1 demonstrates that the monotreme and marsupial UCP1 gene is more closely related to that of ectothermic rather than eutherian orthologs, suggesting that monotremes and marsupials may have never evolved a thermogenic competent UCP1 (Oelkrug et al., 2015). The success of eutherian species radiation and niche expansion has been linked to BAT based thermogenesis (Cannon and Nedergaard, 2004; Oelkrug et al., 2015), although Cannon and Nedergaard (2004) clearly overstate the case when they claim that brown fat derived heat is essential for arousal from hibernation in mammals: it may be essential for eutherian hibernators but echidnas and a number of marsupial hibernators (Geiser and Körtner, 2010), with no BAT, arouse from hibernation quite successfully. Augee and Ealey (1968) reported rewarming rates of echidnas to be lower than for other hibernators. Geiser and Baudinette (1990) demonstrated that in mammals rewarming rates were inversely related to body mass but did not find any difference between the rates of rewarming for marsupials and eutherian mammals. The largest marsupial that shows deep hibernation is the mountain pygmy possum (*Burramys parvus*), which weighs less than 70 g (Ruf and Geiser, 2015), which means there were no marsupials of equivalent size to the echidna and marmot to include in this comparison. In large hibernators rewarming follows a sigmoidal trajectory (Nicol et al., 2009) and rewarming rate varies with T_b_. Figure 6 shows peak rewarming rates for marmots, which have significant amounts of BAT, and for echidnas of the same body mass. Not only are rewarming rates of echidnas much lower than those of marmots, but the relationships between T_b_ and rewarming rate are very different: marmots at low T_b_ have higher maximal rewarming rates than marmots at higher T_b_, while echidnas with a lower T_b_ have lower peak rewarming rates than warmer echidnas. BAT appears to offer a far superior mechanism for rewarming from very low T_b_.

## Monotremes and the evolution of endothermy

There have been numerous models proposed for evolution of endothermy; of these three have received recent attention because they are physiologically testable in modern mammals such as monotremes (Lovegrove, 2012). The aerobic scope hypothesis supposes that the evolution of endothermy was driven by selection for enhanced aerobic capacity to support sustained locomotor activity. In the original formulation of this Bennett and Ruben (1979) argued that a warmer body and endothermy were secondary consequences of selection for enhanced aerobic scope. Clarke and Pörtner (2010) have proposed a modification of the aerobic scope hypothesis in which the increase in aerobic scope was achieved through an increase in T_b_. The parental care model proposes that endothermy may have arisen as a consequence of selection for parental care, because endothermy allows a parent to control incubation temperature, facilitating embryonic development. Higher temperatures speed embryonic development but the costs of this extra thermogenesis would have selected for an increase in aerobic capacity (Farmer, 2000; Farmer and Losos, 2003). The assimilation capacity model (Koteja, 2000) is a variation on the parental care model. It argues that the evolution of endothermy in birds and mammals was driven by two factors: (i) a selection for intense post-hatching parental care, particularly feeding offspring, and (ii) the high cost of maintaining the increased capacity of the visceral organs necessary to support high rates of total daily energy expenditures. What can the monotremes bring to this debate? Although it is now understood that the monotremes do not represent an intermediate step on the way to true endothermy they may still provide some insights into its evolution.

Grigg et al. (2004) suggest that short-beaked echidnas have some of the attributes of a protoendotherm: they are relaxed about maintaining a stable T_b_, with large daily cycles of T_b_ (2–5°C) associated with activity, they show a continuum from daily torpor to long-term torpor or hibernation, which is interrupted by periodic arousals, and they may abandon their normal daily pattern with short periods of torpor at any time of the year. Grigg et al. (2004) stress that this does not mean that the short-beaked echidna displays a primitive or inadequate thermoregulatory ability. Rather they suggest it has retained a plesiomorphic condition to tolerate low T_b_. Lovegrove (2012) uses the term protoendotherm to describe mammals such as the echidna which he suggests have retained Cretaceous basoendothermy. His plesiomorphic-apomorphic endothermy (PAE) model suggests that Cretaceous mammals may not have maintained a constant T_b_ throughout the year and daily torpor and hibernation in certain extant stem tropical mammals is a plesiomorphic condition. Heterothermy in protoendotherms might be considered to be the non-adaptive plesiomorphic state, and periodic normothermy, for example during breeding, as is seen in the echidna, is the adaptive state. In this model, highly seasonal, well-regulated adaptive hibernation in high latitude mammals is a derived state of heterothermy (Lovegrove, 2012).

Leaving aside the difficulty in explaining how torpor in birds and mammals could be derived from the most recent common amniote ancestor, an ectotherm which lived 325 million years ago (Shedlock and Edwards, 2009), it is reasonable to assume that early mammals had a low and variable T_b_, and that the low T_b_ of monotremes (31–32°C), the lowest of any of the mammalian orders (Clarke and O'Connor, 2014), reflects an ancestral condition. However, torpor and hibernation in echidnas is extremely variable between geographic sub-species. In warm climates there seems to be a protoendotherm-like continuum from daily torpor to hibernation, while in cooler areas the expression of hibernation is indistinguishable from Lovegrove's apoendothermic highly seasonal, well-regulated adaptive hibernation (Lovegrove, 2012).

Echidnas do provide some useful insight into the relative merits of the parental care and assimilation capacity models. Very close regulation of maternal T_b_ during egg-incubation (Beard et al., 1992; Nicol and Andersen, 2006) is consistent with the parental care model as the maintenance of a high and constant temperature must be energetically expensive. The 10–11 day period of egg-incubation corresponds with the period of organogenesis and neurulation in echidnas (Werneburg and Sánchez-Villagra, 2011). These developmental processes are particularly temperature sensitive (Andrews, 2004), and in reptiles and birds embryonic development is very sensitive to variations in temperature as well as the absolute temperature (Shine, 2005; Du and Shine, 2015). As decreased mortality early in life results in a larger gain in Darwinian fitness than can be achieved by a comparable decrease of mortality at an older age (Stearns, 1992), there would be very strong selection for higher energy expenditure during egg-incubation. By contrast, the fact that the metabolic rate of lactating female echidnas is not measurably higher that of non-lactating females, suggests that post-hatching energy expenditure may not necessarily be as strong a selection force as is suggested by the assimilation-capacity model (Koteja, 2000).

The monotremes also provide some support for the proposal that the increase in aerobic scope in endotherms was achieved through an increase in T_b_ (Clarke and Pörtner, 2010). The very similar maximum metabolic rates of the echidna and platypus, despite their differences in BMR, points to a T_b_ limitation on metabolic capacity. The more energetically expensive lifestyle and higher BMR of the platypus is associated with thyroid hormone levels which exceed those of the majority of eutherian species. Is the monotreme mitochondrial machinery idling at a much higher rate in the platypus but with still the same temperature limited maximal output as echidnas?

The egg-laying mode of reproduction of the monotremes led early researchers to perceive them as living fossils whose physiology will give insights into the physiology of early mammals. Extant monotremes however are highly specialized, and aspects of their physiology are likely to be strongly affected by their ecological niche. They also have a large brain which accounts for about 9% of resting metabolism, which is certainly not a primitive trait. Monotremes show a mosaic of derived and plesiomorphic features in their embryology and development (Werneburg and Sánchez-Villagra, 2011), adult anatomy (Crompton and Jenkins, 1973) and genome (Warren et al., 2008), reinforcing the picture that mammalian evolution is not a story of linear progress starting with monotremes, passing through marsupials and reaching placentals (Werneburg and Sánchez-Villagra, 2011). This mosaic also clearly extends to energetics and thermoregulation.

## Author contributions

The author confirms being the sole contributor of this work and approved it for publication.

## Funding

This work was supported by the Australian Research Council and the National Geographic Committee for Research and Exploration.

### Conflict of interest statement

The author declares that the research was conducted in the absence of any commercial or financial relationships that could be construed as a potential conflict of interest.
